# The retinal ganglion cell layer reflects neurodegenerative changes in cognitively unimpaired individuals

**DOI:** 10.1186/s13195-022-00998-6

**Published:** 2022-04-21

**Authors:** Alicia López-de-Eguileta, Sara López-García, Carmen Lage, Ana Pozueta, María García-Martínez, Martha Kazimierczak, María Bravo, Juan Irure, Marcos López-Hoyos, Pedro Muñoz-Cacho, Noelia Rodríguez-Perez, Diana Tordesillas-Gutiérrez, Alexander Goikoetxea, Claudia Nebot, Eloy Rodríguez-Rodríguez, Alfonso Casado, Pascual Sánchez-Juan

**Affiliations:** 1 Department of Ophthalmology, ‘Marqués de Valdecilla’ University Hospital, Institute for Research ‘Marqués de Valdecilla’ Santander, University of Cantabria, Santander, Spain; 2 Cognitive Impairment Unit, Neurology Service and Centro de Investigación Biomédica en Red sobre Enfermedades Neurodegenerativas (CIBERNED), ‘Marqués de Valdecilla’ University Hospital, Institute for Research ‘Marqués de Valdecilla’ (IDIVAL), University of Cantabria, Santander, Spain; 3 Department of Immunology, ‘Marqués de Valdecilla’ University Hospital of Cantabria, Institute for Research ‘Marqués de Valdecilla’, Santander, Spain; 4 Department of Medicina Familiar y Comunitaria, IDIVAL, Santander, Spain; 5 Valdecilla Biomedical Research Institute (IDIVAL), Santander, Spain; 6 MARBEC Univ Montpellier, CNRS, Ifremer, IRD, Palavas-les-Flots, France

**Keywords:** Alzheimer’s disease, Optical coherence tomography, Amyloid, Tau, Ganglion cell layer, Retinal nerve fiber layer, Neurodegeneration, Hippocampal volume

## Abstract

**Background:**

To evaluate a wide range of optical coherence tomography (OCT) parameters for possible application as a screening tool for cognitively healthy individuals at risk of Alzheimer’s disease (AD), assessing the potential relationship with established cerebrospinal fluid (CSF) core AD biomarkers and magnetic resonance imaging (MRI).

**Methods:**

We studied 99 participants from the Valdecilla Study for Memory and Brain Aging. This is a prospective cohort for multimodal biomarker discovery and validation that includes participants older than 55 years without dementia. Participants received a comprehensive neuropsychological battery and underwent structural 3-T brain MRI, lumbar puncture for CSF biomarkers (phosphorylated-181-Tau (pTau), total Tau (tTau), beta-amyloid 1–42 (Aβ 1–42), and beta-amyloid 1–40 (Aβ 1–40)). All individuals underwent OCT to measure the retinal ganglion cell layer (GCL), the retinal nerve fiber layer (RFNL), the Bruch’s membrane opening-minimum rim width (BMO-MRW), and choroidal thickness (CT). In the first stage, we performed a univariate analysis, using Student’s *t*-test. In the second stage, we performed a multivariate analysis including only those OCT parameters that discriminated at a nominal level, between positive/negative biomarkers in stage 1.

**Results:**

We found significant differences between the OCT measurements of pTau- and tTau-positive individuals compared with those who were negative for these markers, most notably that the GCL and the RNFL were thinner in the former. In stage 2, our dependent variables were the quantitative values of CSF markers and the hippocampal volume. The Aβ 1–42/40 ratio did not show a significant correlation with OCT measurements while the associations between pTau and tTau with GCL were statistically significant, especially in the temporal region of the macula. Besides, the multivariate analysis showed a significant correlation between hippocampal volume with GCL and RNFL. However, after false discovery rate correction, only the associations with hippocampal volume remained significant.

**Conclusions:**

We found a significant correlation between Tau (pTau) and neurodegeneration biomarkers (tTau and hippocampus volume) with GCL degeneration and, to a lesser degree, with damage in RFNL. OCT analysis constitutes a non-invasive and unexpensive biomarker that allows the detection of neurodegeneration in cognitively asymptomatic individuals.

**Supplementary Information:**

The online version contains supplementary material available at 10.1186/s13195-022-00998-6.

## Background

To date, there is no disease-modifying treatment for Alzheimer’s disease (AD). In particular, clinical trials with anti-amyloid drugs have consistently failed to show efficacy in clinical endpoints. However, it is likely that these studies have been conducted in individuals in advanced stages of the disease, in whom lowering amyloid levels might not be enough to halt the disease progression [1]. There is a consensus in the field that clinical trials with potentially disease-modifying treatments should be performed in the early stages of AD. Therefore, it is necessary to develop tests to identify those individuals who are asymptomatic or very mildly symptomatic, but who have a high risk of progressing to cognitive impairment.

The core biomarkers used in AD research can be divided into three categories: (1) biomarkers of beta-amyloid (Aβ) brain deposition: high ligand retention on amyloid positron emission tomography (PET) or low cerebrospinal fluid (CSF) beta-amyloid 1-42 (Aβ 1–42) and Aβ 1–42/40 ratio [2–4]; (2) biomarkers of AD-associated Tau pathology: elevated CSF phosphorylated Tau (pTau) and Tau-PET [4, 5], and (3) biomarkers of neurodegeneration or neuronal injury: CSF total Tau (tTau), ^18^F-fluorodeoxyglucose (FDG)-PET hypometabolism, and atrophy on structural magnetic resonance imaging (MRI) [5, 6]. Advances in the technique and standardization of CSF biomarkers and the emergence of amyloid and Tau-PET have considerably improved the ability to detect preclinical individuals with AD pathological changes and currently are considered as gold standard tests [7–10]. However, despite the increasing attempts to integrate biomarkers into clinical decision-making, diagnosis supported by them is still considered appropriate only for research-related purposes [11]. The clinical environment has not yet evolved properly for this to occur due to challenges with cost, standardization, and accessibility [12, 13]. Therefore, there is an urgent need to develop non-invasive, affordable, and scalable biomarkers. The aim would be to detect preclinical individuals at high risk of cognitive deterioration, as well as to allow monitoring of the effects of disease-modifying treatments.

The retina, a developmental outgrowth of the brain, is considered a window to study disorders in the central nervous system [14–16]. The link between the eye and AD has been established clinically, histologically, and through technological devices such as optical coherence tomography (OCT) [17]. OCT provides a cross-sectional structure of the retina with extremely high resolution, typically on the micrometer scale. The main advantages of OCT for diagnosing probable AD include its non-invasive nature, its wide availability, and the resulting retinal images, which can be analyzed both objectively and qualitatively [18]. There is growing evidence supporting the incorporation of OCT technology into clinical settings managing neurological diseases [19–21]. Currently, the identification of retinal biomarkers in AD using OCT remains an area of active research, and a growing number of studies indicate that OCT reflects AD pathology in individuals with dementia and those in prodromal stages [22–28]. However, few studies have assessed the role of OCT as an AD biomarker in preclinical individuals [22, 29, 30], and even fewer have used CSF AD biomarkers as a gold standard [29]. Based on the above evidence, we propose to evaluate OCT for its potential applications as a population screening tool. The objective of the present study is to explore a wide range of OCT parameters in a well-phenotyped group of community dwellers. OCT measurements included were the retinal ganglion cell layer (GCL) thickness, the peripapillary retinal nerve fiber layer (RFNL) thickness, the Bruch’s membrane opening-minimum rim width (BMO-MRW), and the choroidal thickness (CT). We evaluated their potential relationships with established CSF core AD biomarkers (tTau, pTau, Aβ 1–42, and Aβ 1–40) and MRI (hippocampal volume).

## Methods

### Participants

We included participants from the Valdecilla Study for Memory and Brain Aging recruited between July 2018 and February 2020 at the University Hospital Marqués de Valdecilla (UHMV) in Santander, Spain. This is a prospective study for multimodal biomarker discovery and validation that includes community dwellers older than 55 years. A comprehensive neuropsychological battery, which comprises the main cognitive domains (memory, language, praxis, visual perception, and executive function), was administered by two trained neuropsychologists (AP, MGM). All participants underwent structural 3-T brain MRI, blood draw to obtain DNA and plasma samples, lumbar puncture for CSF biomarkers, and ophthalmological evaluation. Subjects with a history of dementia, neurological or psychiatric disorders, any significant systemic illness, or current use of any medications known to affect cognition were excluded. Ophthalmological exclusion criteria were a refractive error > 6.0 or < 6.0 diopters (D) of spherical equivalent or 3.0 D of astigmatism, any history or showing evidence of ocular surgery or ocular disease, best-corrected visual acuity as poor as 20/40, intraocular pressure (IOP) > 18 mmHg, or history of raised IOP. Similarly, other exclusion criteria included clinically relevant opacities of the optic media and low-quality images due to unstable fixation.

The study protocol and the written consent were approved by the Ethics Committee of the UHMV (reference number 2018.111), and it was performed following the principles of the Declaration of Helsinki. Written consent forms were signed by all participants before examinations.

### Neuropsychological episodic memory testing

To evaluate the medial temporal lobe (MTL) function, the verbal episodic memory of all participants was assessed using the Free and Cued Selective Reminding Test (FCSRT; Buschke, 1984). We used FCSRT delayed total recall as our main variable for episodic memory (0–16).

### CSF samples acquisition and analysis

The CSF biomarker assessment included the determination of Aβ 1–42, Aβ 1–40, tTau, and p-181-Tau. The levels of biomarkers were quantified by chemiluminescent enzyme-immunoassay (Lumipulse G600 II, Fujirebio Europe, Belgium) following the manufacturer’s instructions and interpreted according to the previously established cutoff points [31]. To adjust for individual differences in total amyloid production, Aβ42 was expressed relative to Aβ40 (ratio Aβ 1–42/40).

### Magnetic resonance imaging

All images were acquired in the same 3T Philips Medical Systems MRI scanner (Achieva, Best, The Netherlands) using an 8-channel head coil at the UHMV. A sagittal MPRAGE T1-weighted sequence was acquired with the following parameters: flip angle 9° shortest TR and TE, voxel size = 1.2 mm, and 170 contiguous slices.

To segment the hippocampus, the automated FreeSurfer protocol was used (FreeSurfer version 6.0 (http://surfer.nmr.mgh.harvard.edu)). Briefly, the protocol included the removal of non-brain tissue, labeling volumes of each segmentation, and normalizing the voxel intensities. Next, cortical and subcortical volume measures were inferred using the surface stream and the subcortical segmentation stream, respectively [32].

Subcortical measures were automatically derived from the subcortical processing stream (i.e., “aseg.stats” in FreeSurfer). Quality checks of acquired data were conducted using the ENIGMA Consortium quality control protocol (http://enigma.ini.usc.edu/).

### Ophthalmological assessment

All participants underwent a thorough ophthalmologic examination on the day of OCT imaging: best-corrected visual acuity (Snellen charts), anterior segment biomicroscopy, refraction, OCT measurements, axial length (AL) assessment, IOP quantification with Goldmann applanation tonometer (GAT), and dilated fundus examination. Participants received one drop of tropicamide 1% and phenylephrine per eye for pupil dilation after OCT evaluation to preclude modifications in choroidal thickness due to phenylephrine instillation as has been previously reported [33]. The refractive error was recorded using an auto refractometer Canon RK-F1 (Canon USA Inc., Lake Success, NY, USA). AL was measured using a Lenstar LS 900 (Haag Streit AG, Koeniz, Switzerland). Each individual was randomized to decide which eye was to be examined first, using the method described by Dulku et al. [34].

### Spectral-domain OCT imaging

A well-trained ophthalmologist (ALE) performed all OCT exams of each eye for each patient using spectral-domain OCT (SD-OCT) (Spectralis, Heidelberg Engineering, Heidelberg, Germany) and checked all images from each eye to identify any segmentation or centering errors.

#### Ganglion cell layer thickness

**Fig. 1:**
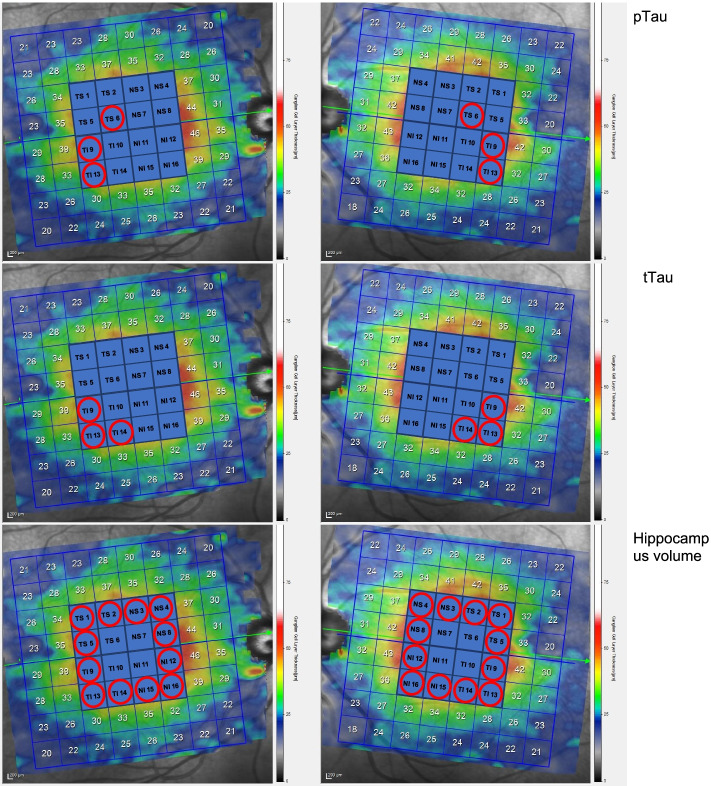
Representation of the optical coherence tomography (OCT) scan of the macula with posterior pole analysis (PPA). PPA is divided into 16 sectors numbered from 1 to 16. Temporal sectors included TS1, TS2, TS5, TS6, TI9, TI10, TI13, and TI14; nasal sectors are NS3, NS4, NS7, NS8, NI11, NI12, NI15, and NI16. Superior sectors were labeled from 1 to 8, whereas inferior sectors were from 9 to 16. The first PPA analysis is related to CSF pTau, the second image is related to CSF tTau, and the last PPA is related to hippocampus volume. Red circles highlight the GCL sectors with significant damage. CSF, cerebrospinal fluid; GCL, ganglion cell layer

The retinal thickness was measured with posterior pole analysis (PPA) software of SD-OCT. This protocol has been described in detail [35]. The average retinal layer measurement of each 8 × 8 (3° × 3°) sector (64 sectors) was determined. To simplify the study, we considered 4 × 4 central grids for analysis. Those 16 sectors were numbered as shown in Fig. 1, with temporal (T), nasal (N), superior (S), and inferior (I) labels added to ease understanding. The superior cluster included 1–8 sectors, whereas the inferior cluster included 9–16 sectors (Fig. 1). A segmentation analysis was performed using the Heidelberg segmentation software (version 1.10.2.0) to calculate the thickness of the GCL considering APOSTEL recommendations [36].

#### Peripapillary retinal nerve fiber layer thickness

The “Glaucoma Module Premium Edition” (GMPE) provided by Spectralis version 6.0c was used to evaluate optic nerve variations in AD. This technology has proven useful in glaucoma disease [37] and also for evaluating optic discs with anatomical variations [38]. The BMO-MRW study is widely used in glaucoma but not in neurodegenerative diseases, so we performed it for two reasons. On the one hand, we employed it to detect and exclude participants with glaucoma disease. On the other hand, we wanted to provide a more in-depth analysis of the optic nerve in AD because damage to the peripapillary axons of the optic nerve in advanced stages of AD has been widely described [39], but there are some controversial results in the prodromal stages [22, 29, 30].

GMPE includes 24 radial scans for the neuroretinal rim analysis (BMO-MRW) and 3 circular scans for the RNFL analysis [40]. From the 3 circular scans, we registered only the figures provided by the inner circle scan. Six sector areas (superior-temporal (ST), superior (S), superior-nasal (SN), inferior-nasal (IN), inferior (I), and inferior-temporal (IT)), as well as their average, were measured in both analyses.

#### Choroidal thickness

CT was measured using enhanced depth imaging (EDI) Spectralis SD-OCT. CT was measured at 14 different locations as it has been previously described [25]: at the fovea (with horizontal and vertical scan: FH and FV, respectively) and at 500, 1000, and 1500 μm from the fovea in the N, T, S, and I quadrants.

### Data analysis

In the first stage of the analysis, we dichotomized all individuals according to their CSF AD biomarker status using previously established cutoff points [31]. Next, potential confounders were evaluated by comparing ophthalmological variables (IOP, pachymetry, AL, and refractive error) in individuals with positive versus negative AD biomarkers. For each individual, we calculated the mean value of the two eyes for all OCT measurements (GCL, RNFL, BMO-MRW, and CT). Then, we performed a univariate analysis using Student’s *t*-test, comparing OCT parameters in those individuals positive for AD CSF biomarkers versus those who were negative.

In a second stage, we performed multivariate analyses including only those OCT parameters that discriminated, at a nominal level, between individuals with positive versus negative biomarkers in stage 1. We used generalized linear models with the biomarkers’ quantitative data as dependent variables (Aβ 1–42/40 ratio, pTau, tTau, and hippocampus volume) and the OCT measurements, selected after stage 1, as the main independent variables.

Age and sex were included as covariates in all models. In the second stage, we determined the false discovery rate (FDR) using the Benjamini and Hochberg method to correct for multiple testing due to the many retinal areas explored [41].

Finally, we assessed the relationship between significant OCT parameters and FCSRT delayed total recall using Pearson’s *r*.

All analyses were performed using IBM SPSS Statistics V.20.0 (International Business Machine Corporation, Armonk, NY, USA).

## Results

Overall, 99 individuals (191 eyes) were consecutively evaluated in the final analysis. Females were predominant (71%), and the mean age was 64.7 ± 6.3 years. The demographic and clinical characteristics of the study participants are summarized in Table 1. The majority were cognitively healthy (the mean Mini-Mental State Examination score was 28.94 ± 1.3), and the mean FCSRT delayed total recall was 15.01 ± 2.43.

**Table 1:** Demographic and clinical characteristics

Characteristic	*N* = 99 individuals
Age (years), mean (SD)	64.9 (6.6)
Female eyes (%)	71%
Spherical equivalent, mean (SD)	0.34 (2.04)
Axial length (mm), mean (SD)	23.18 (1.4)
Intraocular pressure, mean (SD)	13.62 (2.77)
Pachimetry, mean (SD)	28.97 (1.21)
MMSE (0–30), mean (SD)	28.94 (1.3)
FCSRT delayed total recall (0–16), mean (SD)	15.01 (2.43)
CSF Biomarkers	
Aβ40, mean (SD), pg/ml	10,615.01 (3278.23)
Aβ42, mean (SD), pg/ml	819.78 (336.35)
Ratio Aβ 1–42/40, mean (SD)	0.078 (0.02)
Total Tau, mean (SD), pg/ml	330.70 (137.25)
Phosphorylated Tau, mean (SD)	43.97 (26.35)
Hippocampal volume, mean (SD)	3266.08 (372.21)

*Abbreviations*: *SD* Standard deviation; *Aβ* Amyloid-β, *neg*, negative; *pos* Positive, *FCSRT* Free and Cued Selective Reminding Test

Ophthalmological examination parameters did not show significant associations with CSF AD biomarkers (Additional file 1). Subsequently, we did not consider them as confounding variables for the analysis.

### Stage 1

**Fig. 2:**
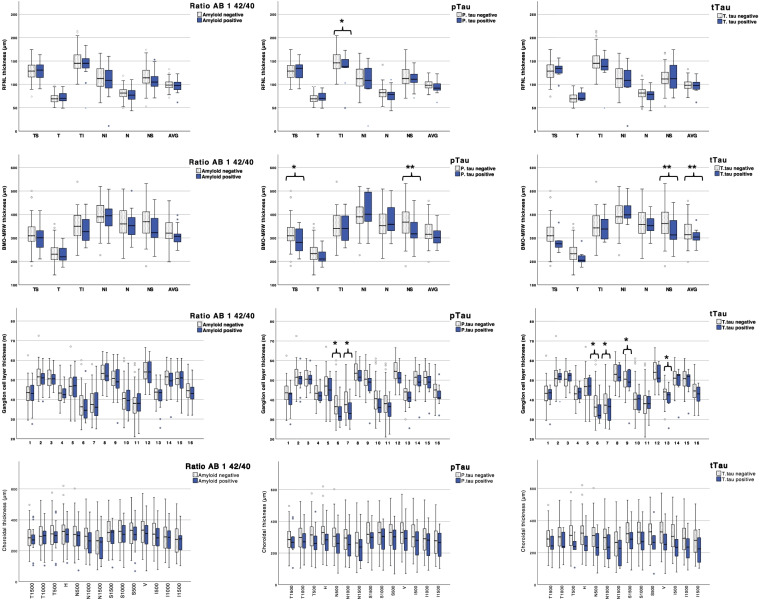
A representation of the outcomes from the univariant analysis through bar charts. Each OCT parameter was compared with the three AD core biomarkers, Aβ 1–42/40 ratio, pTau, and tTau, and divided into healthy and disease cases by well-known cut-points. * and ** symbols highlight the outcomes with significant *p*-values (*p* < 0.05 and *p* < 0.01, respectively). BMO-MRW, Bruch’s membrane opening-minimum rim width; RNFL, retinal nerve fiber layer; GCL, ganglion cell layer; TS, temporal superior; T, temporal; TI, temporal inferior; NI, nasal inferior; N, nasal; NS, nasal superior; AVG, average

Outcomes from stage 1 are depicted in Fig. 2. Herein, we compared OCT parameters (RFNL, GCL, CT, and BMO-MRW) between individuals with positive versus negative AD core biomarkers. None of the OCT measurements significantly discriminated between individuals positive versus negative for the Aβ 1–42/40 ratio. Meanwhile, there were several OCT variables, especially those related to GCL, which significantly differed between positive and negative individuals for pTau and tTau CSF biomarkers. We consistently found that in those individuals who were positive for either pTau or tTau, GCL thickness was decreased compared to those who were negative. We found nominally significant differences between pTau-positive and pTau-negative individuals for GCL in 6 TS (*p* = 0.044) and 7 NS (*p* = 0.043) sectors. Additionally, GCL significantly discriminated between tTau-positive and tTau-negative in 6 TS (*p* = 0.049), 7 NS (*p* = 0.035), 9 TI (*p* = 0.046), and 13 TI (*p* = 0.021) sectors. Overall, individuals with positive pTau and tTau biomarkers showed, on average, lower values on the other OCT variables. Significant differences were found between pTau-positive and pTau-negative individuals for BMO-MRW in TS (*p* = 0.03) and NS (*p* = 0.014) sectors and RFNL in the TI sector (*p* = 0.049). We also found significant differences between tTau-positive and tTau-negative individuals for BMO-MRW NS (*p* = 0.016) and BMO-MRW average (*p* = 0.002).

Choroidal thickness was not associated with any of the CSF biomarkers.

### Stage 2

In stage 2, we performed a multivariate analysis with the OCT measurements that were associated with CSF markers positivity in stage 1, adjusting for age and sex (Table 2).

**Table 2:** Multivariate analysis including all OCT outcomes statistically significant in the univariate analysis

		Ratio AB 1–42/40		pTau		tTau		Hippocampal volume	
		Beta (95%CI)	***p***-value	Beta (95%CI)	***p***-value	Beta (95%CI)	***p***-value	Beta (95%CI)	***p***-value
**BMO-MRW**	**TS**	3.22E−5 (− 5.12E−5 to 11.6E−5)	0.444	− 0.07 (− 0.16 to 0.02)	0.111	− 042 (− 0.92 to 0.07)	0.092	0.26 (− 1.03 to 1.56)	0.688
	**T**	2.82E−5 (− 8.35E−5 to 14.0E−5)	0.503	− 0.02 (− 0.14 to 0.11)	0.812	− 0.14 (− 0.81 to 0.54)	0.687	0.40 (− 1.36 to 2.16)	0.651
	**TI**	3.16E−5 (− 5.08E−5 to 11.4E−5)	0.448	0.01 (− 0.08 to 0.10)	0.800	− 0.01 (− 0.51 to 0.49)	0.968	0.49 (− 0.80 to 1.79)	0.448
	**NI**	2.4E−5 (− 4.84E−5 to 9.71E−5)	**0.006**	0.02 (− 0.06 to 0.10)	0.672	0.07 (− 0.37 to 0.51)	0.756	0.80 (− 0.32 to 1.92)	0.158
	**N**	4.04E−5 (− 3.06E−5 to 11.1E−5)	0.261	− 0.03 (− 0.11 to 0.05)	0.425	− 0.21 (− 0.63 to 0.22)	0.336	0.99 (− 0.09 to 2.07)	0.071
	**NS**	6.64E−5 (− 3.06 to 13.5E−5)	0.056	− 0.07 (− 0.15 to 0.01)	0.073	− 0.56 (− 0.78 to 0.07)	0.099	0.76 (− 0.37 to 1.89)	0.183
**RNFL**	**TS**	− 9.23E−5 (− 32.5E−5 to 14.1E−5)	0.434	0.08 (− 0.18 to 0.34)	0.561	0.49 (− 0.90 to 1.88)	0.485	1.18 (− 2.63 to 4.98)	0.539
	**T**	− 13.8E−5 (− 0.001 to 27.1E−5)	0.505	0.32 (− 0.12 to 0.78)	0.150	2.22 (− 0.18 to 4.61)	0.069	− 0.19 (− 7.00 to 6.60)	0.954
	**TI**	7.68E−5 (− 10.7E−5 to 26.1E−5)	0.408	− 0.17 (− 0.37 to 0.04)	0.108	− 0.97 (− 2.05 to 0.11)	0.076	3.77 (0.90 to 6.63)	0.011*
	**NI**	5.71E−5 (− 11.0E−5 to 22.5E−5)	0.500	− 0.15 (− 0.33 to 0.04)	0.120	− 1.02 (− 2.00 to −0.41)	**0.041**	3.38 (0.75 to 6.01)	0.013*
	**N**	5.08E−5 (− 23.7E−5 to 33.8E−5)	0.726	− 0.04 (− 0.36 to 0.28)	0.813	− 0.76 (− 2.47 to 0.94)	0.337	6.81 (2.45 to 11.17)	0.003*
	**NS**	0.00 (− 7.63E−5 to 31.5E−5)	0.228	− 0.10 (− 0.32 to 0.12)	0.364	− 0.71 (− 1.87 to 0.46)	0.232	4.25 (0.96 to 7.54)	0.012*
**CGL**	**TS 1**	− 1.94E−5 (− 0.001 to 0.001)	0.959	− 0.53 (− 1.36 to 0.29)	0.200	− 2.26 (− 6.70 to 2.18)	0.314	17.13 (5.69 to 25.57)	**0.004**
	**TS 2**	− 8.72E−5 (− 0.001 to 0.001)	0.823	− 0.37 (− 1.23 to 0.48)	0.390	− 1.97 (− 6.57 to 2.64)	0.398	15.58 (3.54 to 27.62)	0.012*
	**NS 3**	0.00 (− 0.001 to 0.001)	0.722	− 0.12 (− 1.09 to 0.86)	0.811	− 0.88 (− 6.13 to 4.36)	0.738	17.85 (3.89 to 31.81)	0.013*
	**NS 4**	0.00 (− 0.001 to 0.001)	0.744	− 0.12 (− 1.13 to 0.89)	0.812	− 0.73 (− 6.14 to 4.67)	0.788	17.33 (2.51 to 32.16)	0.023*
	**TS 5**	4.14E−5 (− 0.001 to 0.001)	0.902	− 0.41 (− 1.15 to 0.32)	0.268	− 2.61 (− 6.56 to 1.35)	0.193	14.66 (4.58 to 24.74)	0.005*
	**TS 6**	0.00 (0.000 to 0.001)	0.123	− 0.78 (− 1.49 to −0.07)	**0.032**	− 3.57 (− 7.42 to 0.28)	0.069	4.23 (− 6.25 to 14.70)	0.424
	**NS 7**	0.00 (0.000 to 0.001)	0.193	− 0.65 (− 1.36 to 0.05)	0.070	− 3.25 (− 7.07 to 0.57)	0.094	6.08 (− 4.28 to 16.44)	0.246
	**NS 8**	− 1.56E−5 (− 0.001 to 0.001)	0.971	− 0.44 (− 1.40 to 0.52)	0.361	− 3.08 (− 8.21 to 2.05)	0.235	18.93 (5.68 to 32.19)	0.006*
	**TI 9**	0.00 (0.000 to 0.001)	0.516	− 0.88 (− 1.69 to −0.08)	**0.032**	− 4.76 (− 9.07 to −0.44)	**0.031**	13.26 (1.75 to 24.77)	0.025*
	**TI 10**	0.00 (− 0.001 to 0.001)	0.726	− 0.46 (− 1.21 to 0.28)	0.219	− 1.49 (− 5.51 to 2.54)	0.465	2.79 (− 8.02 to 13.62)	0.608
	**NI 11**	0.00 (0.000 to 0.001)	0.705	− 0.37 (− 1.04 to 0.31)	0.285	− 1.61 (− 5.25 to 2.04)	0.384	4.06 (− 5.70 to 13.82)	0.410
	**NI 12**	0.00 (− 0.001 to 0.001)	0.480	− 0.69 (− 1.63 to 0.26)	0.150	− 3.87 (− 8.94 to 1.19)	0.132	13.53 (0.01 to 27.05)	**0.050**
	**TI 13**	0.00 (− 0.001 to 0.001)	0.494	− 1.04 (− 2.05 to −0.02)	**0.045**	− 5.43 (− 10.89 to 0.03)	**0.051**	20.42 (5.70 to 35.14)	0.007*
	**TI 14**	0.00 (− 0.001 to 0.001)	0.567	− 0.81 (− 1.70 to 0.08)	0.073	− 4.65 (− 9.42 to 0.11)	**0.055**	16.39 (3.68 to 29.10)	0.012*
	**TI 15**	3.81E−5 (− 0.001 to 0.001)	0.926	− 0.75 (− 1.65 to 0.14)	0.097	− 4.38 (− 9.17 to 0.41)	0.073	14.24 (1.37 to 27.12)	0.031*
	**TI 16**	0.00 (− 0.001 to 0.001)	0.458	− 0.70 (− 1.65 to 0.25)	0.146	− 3.43 (− 8.54 to 1.69)	0.187	16.04 (2.23 to 29.85)	0.023*

Significative values (*p* < 0.005) are in bold

Significative values after the false discovery rate are distinguished with an asterisk

*Abbreviations*: *BMO-MRW*, Bruch’s membrane opening-minimum rim width; *GCL*, ganglion cell layer; *I*, inferior; *N*, nasal; *RNFL*, retinal nerve fiber layer; *S*, superior; *T*, temporal

Once again, Aβ 1–42/40 ratio did not show significant association with any OCT measurements. The association between pTau and tTau with GCL measurements remained significant. Likewise, pTau CSF levels showed significant association with GCL in 6 TS, 9 TI and 13 TI sectors, and tTau showed significant association with GCL in 9 TI, 13 TI, and 14 TI sectors. Regarding RFNL and tTau, only the NI sector showed a significant association. None of the BMO-MRW assessments was significantly associated with any of the CSF markers. However, after the FDR correction, none of these nominal *p*-values remained significant.

The same multivariate analysis was performed for hippocampal volume. We consistently found that larger hippocampal volumes were significantly associated with greater GCL thickness and vice versa. As shown in Table 2, GCL 1 TS, 2 TS, 3 NS, 4 NS, 5 TS, 8 NS, 9 TI, 12 NI 13 TI, 14 TI, 15 NI, and 16 NI sectors were statistically associated to hippocampal volume. Besides, RFNL displayed significant association in many sectors: TI, NI, N, and NS. No association was found with BMO-MRW measurements. After FDR correction, most RFNL and CGL OCT measurements remained significantly associated with the hippocampal volume (Table 2).

In Table 3 we present the relationship between the average of all studied sectors of the RNFL, the GCL and BMO-MRW, and all AD biomarkers. Larger hippocampal volumes were significantly associated with greater RNFL and GCL thickness measurements.

**Table 3:** Multivariate analysis including the average of OCT outcomes statistically significant in the univariate analysis

	Ratio AB 1 42/40		pTau		tTau		Hippocampal volume	
	Beta (95%CI)	***p***-value	Beta (95%CI)	***p***-value	Beta (95%CI)	***p***-value	Beta (95%CI)	***p***-value
**AVG BMO-MRW**	6.47E−5 (− 2.74E−5 to 15.7E−5)	0.166	− 0.5 (− 0.16 to 0.06)	0.335	− 0.21 (− 0.79 to 0.36)	0.465	1.32 (− 0.16 to 2.80)	0.080
**AVG RNFL**	8.97E−5 (− 29.6E−5 to 47.6E−5)	0.645	− 0.14 (− 0.57 to 0.29)	0.517	− 1.06 (− 3.36 to 1.23)	0.361	8.91 (3.06 to 14.76)	**0.003**
**AVG GCL**	− 0.00 (− 0.001 to 0.001)	0.097	− 0.87 (− 1.89 to 0.16)	0.097	− 4.46 (− 9.99 to 1.06)	0.112	18.04 (3.50 to 32.58)	**0.016**

Significative values (*p* < 0.005) are in bold

*Abbreviations*: *BMO-MRW* Bruch’s membrane opening-minimum rim width, *GCL* Ganglion cell layer, *I* Inferior, *N* Nasal, *RNFL* Retinal nerve fiber layer, *S* Superior, *T* Temporal

In Table 4, the GCL data is grouped into 4 main sectors (TS, NS, TI, and NI) to obtain a summary view. Each contains the mean thickness of each area (mean of both eyes). TS is the compound of the mean thickness of sectors 1, 2, 5, and 6; NS is the compound of 3, 4, 7, and 8 sectors; TI is the compound of 9, 10, 13, and 14 sectors; and NI is the compound of 11, 12, 15, and 16 sectors. The TI sector shows nominal associations with all Tau and hippocampal volume markers. After FDR correction, the associations with the hippocampal volume remained significant.

**Table 4:** Multivariate analysis including ganglion cell layer analysis clustered in 4 sectors

	Ratio AB 1 42/40		pTau		tTau		Hippocampal volume	
	Beta (95%CI)	***p***-value	Beta (95%CI)	***p***-value	Beta (95%CI)	***p***-value	Beta (95%CI)	***p***-value
**GCL TS**	1.86E−4 (− 0.001 to 0.001)	0.655	− 0.74 (− 1.64 to 0.17)	0.109	− 3.66 (− 8.54 to 1.21)	0.139	16.73 (4.10 to 29.37)	0.010*
**GCL NS**	1.45E−4 (− 0.001 to 0.001)	0.771	− 0.59 (− 1.67 to 0.50)	0.284	− 3.35 (− 9.17 to 2.48)	0.257	20.15 (4.73 to 35.57)	0.011*
**GCL TI**	2.30E−4 (− 0.001 to 0.001)	0.520	− 1.04 (− 2.03 to − 0.05)	**0.039**	− 5.20 (− 10.52 to 0.13)	**0.050**	15.89 (1.70 to 30.09)	0.029*
**GCL NI**	2.54E−4 (− 0.001 to 0.001)	0.582	− 0.83 (− 1.83 to 0.17)	0.103	− 4.32 (− 9.70 to 1.07)	0.115	14.69 (0.38 to 29.01)	0.044*

Significative values (*p* < 0.005) are in bold

Significative values after the false discovery rate are distinguished with an asterisk

*Abbreviations*: *GCL* Ganglion cell layer, *I* Inferior, *N* Nasal, *RNFL* Retinal nerve fiber layer, *S* Superior, *T* Temporal

Finally, the FCSRT delayed total recall did not show any correlation with the OCT parameters.

## Discussion

Our main results show a nominally significant association between Tau (pTau) and both neurodegeneration biomarkers (tTau and, especially, hippocampal volume) and GCL degeneration. To a lesser degree, we detected an association betweent Tau and hippocampal volume with RFNL. Despite the exploratory nature of our design, the consistency of our results is remarkable, as all of the GCL associated areas showed the same pattern: the more tTau or pTau levels in the CSF, the thinner was the GCL layer. The opposite was found for the hippocampal volume: larger hippocampal volumes were associated with thicker GCL layers and vice versa. Moreover, the same patterns were present in the RFNL analysis. However, after adjusting the *p*-values using FDR correction, only the hippocampal volume was associated with GCL and RFNL parameters. In contrast, CT did not show any significant correlation with CSF biomarkers, and BMO-MRW measurements were associated with pTau and tTau status in isolated sectors, but *p*-values did not survive multivariate analysis. Overall, we found that none of the OCT measurements was associated with the amyloid biomarker (CSF Aβ 1–42/40 ratio). Finally, we found no association between the episodic memory test, FCSRT, and any of the OCT biomarkers, which is concordant with the hypothesis that retinal changes precede cognitive problems.

The recent research framework for AD proposes an A/T/N classification system based on biomarkers in living patients, independently of cognitive status: “A” refers to the Aβ biomarker (Aβ PET or CSF), “T” refers to pathologic Tau (CSF pTau or PET-Tau), and “N” refers to neurodegeneration (CSF tTau, FDG-PET, or structural MRI) [42]. This new research framework is based on the rationale that AD is a continuum and the A/T/N classification captures the sequential pathological changes starting with Aβ deposition that would produce Tau pathology and, finally, neurodegeneration. Our results support the concept of AD as a continuous biological process [42, 43]. While no OCT measurements correlated with the Aβ biomarker, changes in the retina became significant in those individuals with positive pTau, especially in relation with positive neurodegeneration biomarkers like tTau and hippocampal atrophy. As shown in Fig. 1, the GCL damage was more extensive in those individuals whose biomarker profile showed a more advanced disease course. Moreover, we found that the correlation between CSF pTau and tTau (6 TS, 9 TI, 13 TI and 9 TI, 13 TI, 14 TI, respectively) with GCL was strongest in the macular peripheral sectors. The same pattern, with even larger effects, was found in the hippocampal volume analysis.

Only a handful of studies have suggested a link between AD biomarkers and retinal OCT measurements in cognitively unimpaired individuals [22, 29, 30]. Particularly, Asanad et al. [29] reported that the mean RFNL was thinner in individuals that had CSF biomarkers of AD pathology before cognitive deficits. Santos et al. [30] also found larger RNFL damage in preclinical AD relative to controls and related this damage to increased neocortical amyloid accumulation detected by ^18^F-florbetapir Aβ PET. In contrast to our study, Santos and co-authors did not find an association between GCL thinning and AD biomarkers [30]. One possible explanation for these conflicting results is that some of the aforementioned studies have less statistical power. While our population included 99 individuals (mean of both eyes, 191 eyes in total), these previous studies evaluated 43 participants and 56 eyes, respectively. In line with our results, Golzan et al. reported a significant difference in GCL thickness between AD, preclinical AD, and healthy controls [22]. However, they found no association between OCT measurements and PET imaging evidence of brain amyloidosis [22]. In comparison, in our study, we used CSF biomarkers, which are known to detect Alzheimer’s pathological changes earlier than PET [44].

Other studies have investigated the relationship between MRI and retinal thickness in cognitively normal subjects, describing the association between gray matter volume or temporal lobe atrophy and retinal layer [28, 45, 46]. Casaletto et al. demonstrated an association between GCL loss and RNFL thinning with MTL atrophy in neurologically normal older adults [45]. Also, in line with our results, another group found that macular GCL and inner plexiform layer (IPL) thinning was associated with lower gray matter volume of the occipital and temporal lobes in elderly subjects [47]. Our results, adjusted by age, show that GCL damage is related not only to hippocampal atrophy but also to increased CSF pTau and tTau, suggesting that the most likely mechanism would be preclinical AD changes. According to this, Bevan et al. [48] reported, in a transgenic AD model, that degeneration in GCL happens simultaneously with the loss of hippocampal dendritic spines. Interestingly, this constitutes a key hallmark of AD research models, with spine loss particularly acute in the vicinity of amyloid plaques [49].

Despite the cross-sectional design, the predominant association between pTau CSF levels and thinning of the macular temporal peripheral sector of GCL would suggest that it is in this area where we might find the earliest AD-related degenerative changes in the retina of cognitively unimpaired individuals. Retinal ganglion cells, neurons located in the retinal GCL (mostly in the macula), are characterized by a soma from which the originating axon runs initially into the RNFL. Subsequently, these axons converge turning into the optic disc. There is increasing evidence describing the existence of AD pathology in the retina of AD patients [17, 50–58], specifically in the GCL [59]. However, the localization of these changes has been controversial. Neuroretinal damage (RFNL or GCL) in the upper, lower, or temporal macular sectors has been described [24, 27, 28, 60, 61], most likely reflecting that the tissues belonged to individuals at different time points in the Alzheimer’s continuum. Koronyo et al studied the distribution of Aβ plaques in the retina, reporting that Aβ deposits were frequently concentrated in the middle and far periphery of the superior quadrants along the blood vessels [17]. In line with our results, a recent study found that the mid-peripheral retina showed more AD pathology than the central retina [62]. Furthermore, this study showed that the temporal retina had the strongest correlations with brain neuritic plaques and cerebral amyloid angiopathy, and this area showed the greatest contrast between AD and controls, leading the authors to suggest that it might be the optimal location for in vivo ocular imaging [62].

## Limitations

A potential technical limitation of our study is the fact that CT thickness was measured manually after the EDI-OCT scan, providing us with a choroidal analysis based on subjective, non-automated measurements. To help overcome this hurdle, we tested the agreement in a control sample, and as shown in a previous publication, we proved to have high intra-observer and inter-observer reproducibilities [25]. A more general limitation of the present study is the relatively small sample size in comparison with the large number of OCT parameters measured, which might increase the probability of false positives. However, with our two-step design, we have sought to minimize the type 1 error. Additionally, we performed a FDR correction to account for multiple testing due to the large number of areas tested. Most importantly, we think that the consistency of the OCT associations across several biomarkers with similar meaning, like tTau and hippocampal volume, makes it highly unlikely that our results are explained by chance alone.

## Conclusions

Our main finding was the significant association between GCL thickness, measured by OCT, with Tau and neurodegeneration biomarkers in cognitively unimpaired individuals. According to our results, macular temporal peripheral sectors of GCL may represent the areas with the greatest clinical potential for early screening; but this hypothesis requires sequential studies in a larger population to evaluate their clinical utility. Even though our study represents the largest cohort of cognitively unimpaired individuals assessed with OCT and multimodal AD biomarkers to date, our results should be considered exploratory. Future investigations including larger samples and analyzing prospectively the spatiotemporal changes of OCT measurements are needed to optimize the diagnostic utility of retinal imaging modalities in the diagnosis of preclinical AD.

## Supplementary Information

**Additional file 1.** Ophthalmological variables analysis as possible confounders. Potential confounders were evaluated by comparing ophthalmological variables (like IOP, pachymetry, AL and refractive error) in individuals with positive versus negative AD biomarkers. These variables did not show significant associations with CSF AD biomarkers.

## Abbreviations

AD: Alzheimer’s disease
Aβ: Beta-amyloid
Aβ 1–42: Beta-amyloid 1–42
BMO-MRW: Bruch’s membrane opening-minimum rim width
CSF: Cerebrospinal fluid
CT: Choroidal thickness
D: Diopters
EDI: Enhanced depth imaging
FDG: ^18^F-fluorodeoxyglucose
FDR: False discovery rate
FCSRT: Free and Cued Selective Reminding Test
GCL: Ganglion cell layer
IN: Infero-nasal
I: Inferior
IT: Infero-temporal
IOP: Intraocular pressure
MRI: Magnetic resonance imaging
UHMV: Marqués de Valdecilla
OCT: Optical coherence tomography
pTau: Phosphorylated Tau
PET: Positron emission tomography
PPA: Posterior pole analysis
RFNL: Retinal nerve fiber layer
ST: Superior-temporal
S: Superior
SN: Superior-nasal
FH: Subfoveal horizontal scan
FV: Subfoveal vertical scan
tTau: Total Tau

## References

[1] Tolar M, Abushakra S, Sabbagh M. The path forward in Alzheimer’s disease therapeutics: reevaluating the amyloid cascade hypothesis. Alzheimer’s Dementia. 2020;16(11):1553–60. 10.1016/j.jalz.2019.09.075 31706733

[2] Visser PJ, Verhey F, Knol DL, Scheltens P, Wahlund L-O, Freund-Levi Y, et al. Prevalence and prognostic value of CSF markers of Alzheimer’s disease pathology in patients with subjective cognitive impairment or mild cognitive impairment in the DESCRIPA study: a prospective cohort study. Lancet Neurol. 2009;8(7):619–27. 10.1016/S1474-4422(09)70139-5 19523877

[3] Fagan AM, Roe CM, Xiong C, Mintun MA, Morris JC, Holtzman DM. Cerebrospinal fluid Tau/β-amyloid42 ratio as a prediction of cognitive decline in nondemented older adults. Arch Neurol. 2007;64(3):343–9. 10.1001/archneur.64.3.noc60123 17210801

[4] Mattsson N, Henrik Z, Hansson O, Andreasen N, Parnetti L, Jonsson M, et al. CSF biomarkers and incipient Alzheimer disease in patients with mild cognitive impairment. JAMA. 2009;302(4):385–93. 10.1001/jama.2009.1064 19622817

[5] Haan J, Kreeke JA, Konijnenberg E, Kate M, Braber A, Barkhof F, et al. Retinal thickness as a potential biomarker in patients with amyloid-proven early- and late-onset Alzheimer’s disease. Alzheimer’s Dementia. 2019;11(1):463–71. 10.1016/j.dadm.2019.05.002 PMC6584766 31249859

[6] Besson FL, la Joie R, Doeuvre L, Gaubert M, Mezenge F, Egret S, et al. Cognitive and brain profiles associated with current neuroimaging biomarkers of preclinical Alzheimer’s disease. J Neurosci. 2015;22, 35(29):10402–11. 10.1523/JNEUROSCI.0150-15.2015 PMC6605120 26203136

[7] Chételat G, la Joie R, Villain N, Perrotin A, de La Sayette V, Eustache F, et al. Amyloid imaging in cognitively normal individuals, at-risk populations and preclinical Alzheimer’s disease. NeuroImage. 2013;2:356–65. 10.1016/j.nicl.2013.02.006 PMC3777672 24179789

[8] Arnerić SP, Batrla-Utermann R, Beckett L, Bittner T, Blennow K, Carter L, et al. Cerebrospinal fluid biomarkers for Alzheimer’s disease: a view of the regulatory science qualification landscape from the coalition against major diseases CSF biomarker team. J Alzheimer’s Dis. 2016;55(1):19–35. 10.3233/JAD-160573 PMC5115607 27662307

[9] Johnson KA, Schultz A, Betensky RA, Becker JA, Sepulcre J, Rentz D, et al. Tau positron emission tomographic imaging in aging and early Alzheimer disease. Ann Neurol. 2016;79(1):110–9. 10.1002/ana.24546 PMC4738026 26505746

[10] Ossenkoppele R, Schonhaut DR, Schöll M, Lockhart SN, Ayakta N, Baker SL, et al. Tau PET patterns mirror clinical and neuroanatomical variability in Alzheimer’s disease. Brain. 2016;139(5):1551–67. 10.1093/brain/aww027 PMC5006248 26962052

[11] Dubois B, Feldman HH, Jacova C, DeKosky ST, Barberger-Gateau P, Cummings J, et al. Research criteria for the diagnosis of Alzheimer’s disease: revising the NINCDS–ADRDA criteria. Lancet Neurol. 2007;6(8):734–46. 10.1016/S1474-4422(07)70178-3 17616482

[12] Tu P, Fu H, Cui M. Compounds for imaging amyloid-β deposits in an Alzheimer’s brain: a patent review. Expert Opinion Therapeut Patents. 2015;25(4):413–23. 10.1517/13543776.2015.1007953 25746836

[13] Khan TK, Alkon DL. Alzheimer’s disease cerebrospinal fluid and neuroimaging biomarkers: diagnostic accuracy and relationship to drug efficacy. J Alzheimer’s Dis. 2015;46(4):817–36. 10.3233/JAD-150238 26402622

[14] Trost A, Lange S, Schroedl F, Bruckner D, Motloch KA, Bogner B, et al. Brain and retinal pericytes: origin, function and role. Frontiers in Cellular Neuroscience. 2016;10:20. 10.3389/fncel.2016.00020 PMC4740376 26869887

[15] Byerly MS, Blackshaw S. Vertebrate retina and hypothalamus development. Wiley Interdisciplin Rev. 2009;1(3):380–9. 10.1002/wsbm.22 20836003

[16] Purves D, Augustine GJ, Fitzpatrick D, Katz LC, LaMantia A-S, McNamara JO (2001). Neuroscience.

[17] Koronyo Y, Biggs D, Barron E, Boyer DS, Pearlman JA, Au WJ, et al. Retinal amyloid pathology and proof-of-concept imaging trial in Alzheimer’s disease. JCI Insight. 2017;2(16):e93621. 10.1172/jci.insight.93621 PMC5621887 28814675

[18] Huang D, Swanson E, Lin C, Schuman J, Stinson W, Chang W, et al. Optical coherence tomography. Science. 1991;254(5035):1178–81. 10.1126/science.1957169 PMC4638169 1957169

[19] Pellegrini M, Vagge A, Ferro Desideri L, Bernabei F, Triolo G, Mastropasqua R, et al. Optical coherence tomography angiography in neurodegenerative disorders. Journal of. Clin Med. 2020;9(6):1706. 10.3390/jcm9061706 PMC7356677 32498362

[20] Zhou W, Tao J, Li J. Optical coherence tomography measurements as potential imaging biomarkers for Parkinson’s disease: a systematic review and meta-analysis. Eur J Neurol. 2021;28(3):763–74. 10.1111/ene.14613 33107159

[21] Vidal-Jordana A, Pareto D, Cabello S, Alberich M, Rio J, Tintore M, et al. Optical coherence tomography measures correlate with brain and spinal cord atrophy and multiple sclerosis disease-related disability. Eur J Neurol. 2020;27(11):2225–32. 10.1111/ene.14421 32602573

[22] Golzan SM, Goozee K, Georgevsky D, Avolio A, Chatterjee P, Shen K, et al. Retinal vascular and structural changes are associated with amyloid burden in the elderly: ophthalmic biomarkers of preclinical Alzheimer’s disease. Alzheimer’s Res Ther. 2017;9(1):13. 10.1186/s13195-017-0239-9 PMC5335799 28253913

[23] Kwon JY, Yang JH, Han JS, Kim DG. Analysis of the retinal nerve fiber layer thickness in Alzheimer disease and mild cognitive impairment. Korean J Ophthalmol. 2017;31(6):548–56. 10.3341/kjo.2016.0118 PMC5726990 29022297

[24] Cheung CY, Ong YT, Hilal S, Ikram MK, Low S, Ong YL, et al. Retinal ganglion cell analysis using high-definition optical coherence tomography in patients with mild cognitive impairment and Alzheimer’s disease. J Alzheimer’s Dis. 2015;45(1):45–56. 10.3233/JAD-141659 25428254

[25] López-de-Eguileta A, Lage C, López-García S, Pozueta A, García-Martínez M, Kazimierczak M, et al. Evaluation of choroidal thickness in prodromal Alzheimer’s disease defined by amyloid PET. PLOS One. 2020;15(9):e0239484. 10.1371/journal.pone.0239484 PMC7505462 32956392

[26] Bulut M, Yaman A, Erol MK, Kurtuluş F, Toslak D, Doğan B, et al. Choroidal thickness in patients with mild cognitive impairment and Alzheimer’s type dementia. J Ophthalmol. 2016;2016:7291257. 10.1155/2016/7291257 PMC4748862 26925259

[27] Choi SH, Park SJ, Kim NR. Macular ganglion cell-inner plexiform layer thickness is associated with clinical progression in mild cognitive impairment and Alzheimers disease. PLoS One. 2016;11(9):e0162202. 10.1371/journal.pone.0162202 PMC5012569 27598262

[28] Liu S, Ong Y-T, Hilal S, Loke YM, Wong TY, Chen CL-H, et al. The association between retinal neuronal layer and brain structure is disrupted in patients with cognitive impairment and Alzheimer’s disease. J Alzheimer’s Dis. 2016;54(2):585–95. 10.3233/JAD-160067 27567815

[29] Asanad S, Fantini M, Sultan W, Nassisi M, Felix CM, Wu J, et al. Retinal nerve fiber layer thickness predicts CSF amyloid/Tau before cognitive decline. PLoS One. 2020;15(5):e0232785. 10.1371/journal.pone.0232785 PMC7259639 32469871

[30] Santos CY, Johnson LN, Sinoff SE, Festa EK, Heindel WC, Snyder PJ. Change in retinal structural anatomy during the preclinical stage of Alzheimer’s disease. Alzheimer’s Dementia. 2018;10(1):196–209. 10.1016/j.dadm.2018.01.003 PMC5956814 29780864

[31] Alcolea D, Pegueroles J, Muñoz L, Camacho V, López-Mora D, Fernández-León A, et al. Agreement of amyloid PET and CSF biomarkers for Alzheimer’s disease on Lumipulse. Annals of Clinical and Translational. Neurology. 2019;6(9):1815–24. 10.1002/acn3.50873 PMC6764494 31464088

[32] Fischl B, Salat DH, Busa E, Albert M, Dieterich M, Haselgrove C, et al. Whole brain segmentation. Neuron. 2002;33(3):341–55. 10.1016/s0896-6273(02)00569-x 11832223

[33] Casado A, López-de-Eguileta A, Gaitán J, Fonseca S, Gordo-Vega MA. Peripapillary and macular choroidal thickness before and after phenylephrine instillation. Eye. 2019;33(11):1741–7. 10.1038/s41433-019-0478-z PMC7002579 31164729

[34] Dulku S. Generating a random sequence of left and right eyes for ophthalmic research. Investigative Opthalmology & Visual. Science. 2012;53(10):6301–2. 10.1167/iovs.12-10737 22993249

[35] Casado A, Cerveró A, López-de-Eguileta A, Fernández R, Fonseca S, González JC (2019). Topographic correlation and asymmetry analysis of ganglion cell layer thinning and the retinal nerve fiber layer with localized visual field defects. PLoS One..

[36] Cruz-Herranz A, Balk LJ, Oberwahrenbrock T, Saidha S, Martinez-Lapiscina EH, Lagreze WA, et al. The APOSTEL recommendations for reporting quantitative optical coherence tomography studies. Neurology. 2016;86(24):2303–9. 10.1212/WNL.0000000000002774 PMC4909557 27225223

[37] Sastre-Ibañez M, Martinez-de-la-Casa JM, Rebolleda G, Cifuentes-Canorea P, Nieves-Moreno M, Morales-Fernandez L (2018). Utility of Bruch membrane opening-based optic nerve head parameters in myopic subjects. Eur J Ophthalmol.

[38] Rebolleda G, Casado A, Oblanca N, Muñoz-Negrete F (2016). The new Bruch’s membrane opening-minimum rim width classification improves optical coherence tomography specificity in tilted discs. Clin Ophthalmol.

[39] Chan VTT, Sun Z, Tang S, Chen LJ, Wong A, Tham CC (2019). Spectral-domain OCT measurements in Alzheimer’s disease: a systematic review and meta-analysis. Ophthalmology..

[40] López-de-Eguileta A, Lage C, López-García S, Pozueta A, García-Martínez M, Kazimierczak M (2019). Ganglion cell layer thinning in prodromal Alzheimer’s disease defined by amyloid PET. Alzheimer’s & Dementia.

[41] Benjamini Y, Hochberg Y (1995). Controlling the false discovery rate: a practical and powerful approach to multiple testing. J Royal Stat Soc Ser B..

[42] Jack CR, Bennett DA, Blennow K, Carrillo MC, Feldman HH, Frisoni GB (2016). A/T/N: an unbiased descriptive classification scheme for Alzheimer disease biomarkers. Neurology..

[43] Jack CR, Knopman DS, Jagust WJ, Petersen RC, Weiner MW, Aisen PS (2013). Tracking pathophysiological processes in Alzheimer’s disease: an updated hypothetical model of dynamic biomarkers. Lancet Neurol.

[44] Luo J, Agboola F, Grant E, Masters CL, Albert MS, Johnson SC, et al. Sequence of Alzheimer disease biomarker changes in cognitively normal adults. Neurology. 2020;95(23):e3104–16. 10.1212/WNL.0000000000010747 PMC7734923 32873693

[45] Casaletto KB, Ward ME, Baker NS, Bettcher BM, Gelfand JM, Li Y, et al. Retinal thinning is uniquely associated with medial temporal lobe atrophy in neurologically normal older adults. Neurobiol Aging. 2017;51:141–7. 10.1016/j.neurobiolaging.2016.12.011 PMC5554591 28068565

[46] Mejia-Vergara AJ, Karanjia R, Sadun AA. OCT parameters of the optic nerve head and the retina as surrogate markers of brain volume in a normal population, a pilot study. J Neurol Sci. 2021;420:117213. 10.1016/j.jns.2020.117213 33271374

[47] Ong Y-T, Hilal S, Cheung CY, Venketasubramanian N, Niessen WJ, Vrooman H, et al. Retinal neurodegeneration on optical coherence tomography and cerebral atrophy. Neurosci Letters. 2015;584:12–6. 10.1016/j.neulet.2014.10.010 25451722

[48] Bevan RJ, Hughes TR, Williams PA, Good MA, Morgan BP, Morgan JE. Retinal ganglion cell degeneration correlates with hippocampal spine loss in experimental Alzheimer’s disease. Acta Neuropathologica. Communications. 2020;8(1):216. 10.1186/s40478-020-01094-2 PMC7720390 33287900

[49] Spires TL. Dendritic spine abnormalities in amyloid precursor protein transgenic mice demonstrated by gene transfer and intravital multiphoton microscopy. J Neurosci. 2005;25(31):7278–87. 10.1523/JNEUROSCI.1879-05.2005 PMC1820616 16079410

[50] Alexandrov PN, Pogue A, Bhattacharjee S, Lukiw WJ. Retinal amyloid peptides and complement factor H in transgenic models of Alzheimer’s disease. NeuroReport. 2011;22(12):623–7. 10.1097/WNR.0b013e3283497334 PMC3719862 21734608

[51] Schön C, Hoffmann NA, Ochs SM, Burgold S, Filser S, Steinbach S, et al. Long-term in vivo imaging of fibrillar Tau in the retina of P301S transgenic mice. PLoS One. 2012;7(12):e53547. 10.1371/journal.pone.0053547 PMC3534024 23300938

[52] Tsai Y, Lu B, Ljubimov AV, Girman S, Ross-Cisneros FN, Sadun AA, et al. Ocular changes in TgF344-AD rat model of Alzheimer’s disease. Investigative Opthalmology & Visual. Science. 2014;55(1):523–34. 10.1167/iovs.13-12888 PMC3907137 24398104

[53] la Morgia C, Ross-Cisneros FN, Koronyo Y, Hannibal J, Gallassi R, Cantalupo G, et al. Melanopsin retinal ganglion cell loss in Alzheimer disease. Ann Neurol. 2016;79(1):90–109. 10.1002/ana.24548 PMC4737313 26505992

[54] Grimaldi A, Brighi C, Peruzzi G, Ragozzino D, Bonanni V, Limatola C, et al. Inflammation, neurodegeneration and protein aggregation in the retina as ocular biomarkers for Alzheimer’s disease in the 3xTg-AD mouse model. Cell Death Dis. 2018;9(6):685. 10.1038/s41419-018-0740-5 PMC5992214 29880901

[55] Hadoux X, Hui F, Lim JKH, Masters CL, Pébay A, Chevalier S, et al. Non-invasive in vivo hyperspectral imaging of the retina for potential biomarker use in Alzheimer’s disease. Nature. Communications. 2019;10(1):4227. 10.1038/s41467-019-12242-1 PMC6748929 31530809

[56] Schultz N, Byman E, Wennström M. Levels of retinal amyloid-β correlate with levels of retinal IAPP and hippocampal amyloid-β in neuropathologically evaluated individuals. J Alzheimer’s Dis. 2020;73(3):1201–9. 10.3233/JAD-190868 PMC7081096 31884473

[57] Shi H, Koronyo Y, Rentsendorj A, Regis GC, Sheyn J, Fuchs D-T, et al. Identification of early pericyte loss and vascular amyloidosis in Alzheimer’s disease retina. Acta Neuropathologica. 2020;139(5):813–36. 10.1007/s00401-020-02134-w PMC7181564 32043162

[58] Koronyo-Hamaoui M, Koronyo Y, Ljubimov A. v., Miller CA, Ko MK, Black KL, et al. Identification of amyloid plaques in retinas from Alzheimer’s patients and noninvasive in vivo optical imaging of retinal plaques in a mouse model. NeuroImage. 2011;54(Suppl 1):S204–17. 10.1016/j.neuroimage.2010.06.020 PMC2991559 20550967

[59] la Morgia C, Ross-Cisneros FN, Sadun AA, Carelli V. Retinal ganglion cells and circadian rhythms in Alzheimer’s disease, Parkinson’s disease, and beyond. Front Neurol. 2017;8:162. 10.3389/fneur.2017.00162 PMC5415575 28522986

[60] Cunha JP, Proença R, Dias-Santos A, Almeida R, Águas H, Alves M, et al. OCT in Alzheimer’s disease: thinning of the RNFL and superior hemiretina. Graefe’s Archive for. Clin Experimental Ophthalmol. 2017;255(9):1827–35. 10.1007/s00417-017-3715-9 28643042

[61] Snyder PJ, Johnson LN, Lim YY, Santos CY, Alber J, Maruff P, et al. Nonvascular retinal imaging markers of preclinical Alzheimer’s disease. Alzheimer’s Dementia. 2016;4(1):169–78. 10.1016/j.dadm.2016.09.001 PMC5078641 27830174

[62] Lee S, Jiang K, McIlmoyle B, To E, Q Alis X, Hirsch-Reinshagen V, et al. Amyloid beta immunoreactivity in the retinal ganglion cell layer of the Alzheimer’s eye. Front Neurosci. 2020;31:14:758. 10.3389/fnins.2020.00758 PMC7412634 32848548

