# Supplementary material for: The retinal ganglion cell layer reflects neurodegenerative changes in cognitively unimpaired individuals
Source: Alzheimers Res Ther. 2022 Apr 21;14:57. doi: 10.1186/s13195-022-00998-6 (PMC9022357; doi:10.1186/s13195-022-00998-6)
Supplement: Supplementary file 1 — Additional file 1. Ophthalmological variables analysis as possible confounders. Potential confounders were evaluated by comparing ophthalmological variables (like IOP, pachymetry, AL and refractive error) in individuals with positive versus negative AD biomarkers. These variables did not show significant associations with CSF AD biomarkers. [file 13195_2022_998_MOESM1_ESM.docx]

**Additional file 1:** Ophthalmological variables analysis as possible confounders.

| **Median** | **Ratio Aβ** | | | **pTau** | | | **tTau** | | |
| --- | --- | --- | --- | --- | --- | --- | --- | --- | --- |
|  | *Positive* | *Negative* | *p* | *Positive* | *Negative* | *p* | *Positive* | *Negative* | *p* |
| **Intraocular pressure** | 14.5 | 13.5 | 0.122 | 14.25 | 13.5 | 0.088 | 14.25 | 13.5 | 0.463 |
| **Axial lenght** | 23.16 | 23.07 | 0.110 | 22.98 | 23.12 | 0.902 | 23.12 | 23.07 | 0.264 |
| **Spherical equivalent** | 0.44 | 0.38 | 0.824 | 0.69 | 0.38 | 0.795 | 0.56 | 0.38 | 0.679 |
| **Pachimetry** | 533.0 | 538.0 | 0.877 | 531.5 | 538.0 | 0.478 | 531.5 | 536 | 0.995 |

Potential confounders were evaluated by comparing ophthalmological variables (like IOP, pachymetry, AL and refractive error) in individuals with positive versus negative AD biomarkers. These variables did not show significant associations with CSF AD biomarkers.

Abbreviations: Aβ, amyloid-β, pTau, phosphorylated tau, tTau), total tau.
